# Atractylenolide II combined with Interferon-γ synergistically ameliorates colorectal cancer progression *in vivo* and *in vitro* by blocking the NF-kB p65/PD-L1 pathway

**DOI:** 10.7150/jca.96647

**Published:** 2024-06-11

**Authors:** Yangsheng Wu, Shijie Dai, YuJia Zhang, Zheming Li, Bo Zhu, Qingsheng Liu, Like Wo, Zhiling Yu, Xiaofeng Yuan, Xiaobing Dou

**Affiliations:** 1 College of Life Science, Zhejiang Chinese Medical University, Hangzhou, Zhejiang, China.; 2 Academy of Chinese Medical Sciences, Zhejiang Chinese Medical University, Hangzhou, Zhejiang, China.; 3 College of pharmacy, Zhejiang Chinese Medical University, Hangzhou, Zhejiang, China.; 4 Hangzhou Traditional Chinese Medicine Hospital, Hangzhou, Zhejiang, China.; 5 The First Affiliated Hospital of Zhejiang Chinese Medical University, Hangzhou, Zhejiang, China.; 6 School of Chinese Medicine, Hong Kong Baptist University, Hong Kong, China.

**Keywords:** Colorectal cancer, Atractylenolide II, Interferon-γ, PD-L1.

## Abstract

**Purpose:** *Atractylodes macrocephala* Koidz is a widely used classical traditional Chinese herbal medicine, that has shown remarkable efficacy in cancers. Colorectal cancer (CRC) is the most common malignant tumor globally. Interferon (IFN)-γ, a prominent cytokine involved in anti-tumor immunity that has cytostatic, pro-apoptotic, and immune-stimulatory properties for the detection and removal of transformed cells. Atractylenolides-II (AT-II) belongs to the lactone compound that is derived from *Atractylodes macrocephala* Koidz with anti-cancer activity. However, whether AT-II combined with IFN-γ modulates CRC progression and the underlying mechanisms remain unclear. The present study aimed to elucidate the efficacy and pharmaceutical mechanism of action of AT-II combined with IFN-γ synergistically against CRC by regulating the NF-kB p65/PD-L1 signaling pathway.

**Methods:** HT29 and HCT15 cells were treated with AT-II and IFN-γ alone or in combination and cell viability, migration, and invasion were then analyzed using Cell Counting Kit-8 (CCK-8) and Transwell assays, respectively. Furthermore, the underlying mechanism was investigated through western blot assay. The role of AT-II combined with IFN-γ on tumor growth and lung metastases was estimated *in vivo*. Finally, the population of lymphocytes in tumor tissues of lung metastatic C57BL/6 mice and the plasma cytokine levels were confirmed by flow cytometry and enzyme-linked immunosorbent assay (ELISA).

**Results:** AT-II or the combination IFN-γ significantly inhibited the growth and migration abilities of CRC cells *in vitro* and *in vivo*. The biological mechanisms behind the beneficial effects of AT-II combined with IFN-γ were also measured and inhibition of p38 MAPK, FAK, Wnt/β-catenin, Smad, and NF-kB p65/PD-L1 pathways was observed. Moreover, AT-II combined with IFN-γ significantly inhibited HCT15 xenograft tumor growth and lung metastases in C57BL/6 mice, which was accompanied by lymphocyte infiltration into the tumor tissues and inflammatory response inactivation.

**Conclusions:** The results showed that the AT-II in combination with IFN-γ could be used as a potential strategy for tumor immunotherapy in CRC. More importantly, the mechanism by which AT-II suppressed CRC progressions was by inhibiting the NF-kB p65/PD-L1 signal pathway.

## Introduction

Colorectal cancer (CRC), which ranks fourth in malignancy-related deaths in China 1, is a highly aggressive and metastatic cancer of the digestive system in which CRC cells invade and metastasize to different tissues without treatment 2, 3. The proliferative potential of CRC-based cells can induce malignant metastasis of the tumor, such as to the liver and lymphoid organs 3. The survival rate of metastatic CRC is relatively unsatisfying and chemotherapeutic agents have historically been employed as the primary defense in the management of metastatic CRC 4. CRC can develop at any age, although it is most common in those over the age of 50 years. Due to the lack of early diagnostic techniques for CRC, a large number of CRC cases have progressed to advanced CRC with strong metastasis at the time of the initial diagnosis 5. Chemotherapy, targeted therapy, and immunotherapy are the most commonly used systemic therapies for CRC patients. Once malignant metastasis occurs, there are fewer and fewer drugs available to treat metastatic cancer cells. Although having demonstrated that these novel therapies have improved overall survival for CRC, existing drugs for the treatment of metastatic CRC were far from satisfactory 6. Therefore, there is an urgent need to develop effective drugs and therapeutic targets for the treatment of CRC.

It is well known that the early stage of tumor invasion is accompanied by regulation of the microenvironment and suppression of anti-tumor immunity 7. During the regulatory process, immune cells are remodeled by the tumor environment to meet the needs of tumor invasion and metastasis. Therefore, enhancing anticancer immunity would be beneficial in the treatment of highly metastatic tumors. Cytokines are small peptides secreted by immune cells that are involved in cell signaling, which can indirectly enhance anti-tumor activity by activating the body's immune system, and some cytokines can also directly inhibit the growth of tumor cells 8. Currently, cytokines with inhibitory effects on gastrointestinal malignant tumors include interleukin (IL), interferon-gamma (IFN-γ), granulocyte-macrophage-colony stimulating factor (GM-CSF), etc. 8, 9. Among them, IFN-γ is a typical anti-cancer cytokine mainly produced by activated T cells and natural killer cells. IFN-γ promotes anticancer immunity by recruiting highly immunogenic cells such as CD4^+^ and CD8^+^ T cells and infiltrating tumor-associated macrophages in the M1 stage to enhance antitumor immune responses 10. However, prolonged exposure to IFN-γ tends to cause tumors to acquire adaptive immune resistance, which is one major barrier to improving immunotherapy in solid tumors 11. The most significant resistance mechanism involves the induction of programmed death-ligand 1 (PD-L1) expression on the tumor surface. Then, PD-L1 binds to the programmed death 1 (PD-1) receptor expressed on the surface of T cells, leading to T cell inactivation and cancer cell immune escape 11, 12. Clinically, high PD-L1 expression is associated with metastasis and poor prognosis 13. Unlike endogenously controlled constitutive PD-L1 14 expression, IFN-γ-induced PD-L1 expression is associated with a rapid response of the p65 subunit in tumor cells to the IFN-γ-stimulated NF-kB signaling pathway 15.

To minimize the side effects of IFN-γ immunotherapy for tumor treatment, IFN-γ therapy is often combined with other treatments, such as chemotherapy 16. Interestingly, several natural products, such as vitamins and their derivatives, are able to reverse IFN-γ-mediated immune resistance and inhibit tumor growth 17, 18. Moreover, a growing number of studies have shown that numerous natural ingredients have anti-CRC activities and can be employed as alternative chemotherapeutic agents for the treatment of CRC 19, 20. Traditional Chinese Medicine (TCM) has a long history of being employed around the world for the treatment of cancers, and natural medicines, particularly botanicals, are still a significant route for new medical research and development. Atractylodes macrocephala (AM, called Baizhu in China), a perennial plant, is the dried root of *Atractylodes macrocephala Koidz,* first appeared in "The Shen Nong Ben Cao Jing", written more than 2000 years ago. AM has a range of chemical components, such as sesquiterpenes and lactones, flavonoids, phenylpropanoids, alkynes and their glycosides, polysaccharides, and so on 21, 22. Modern pharmacological studies have shown that the AM has a variety of pharmacological effects such as anti-inflammatory, anti-tumor, improving gastrointestinal function, regulating the urinary system, improving neurological function, improving immunity, excitation of uterine smooth muscle, lowering blood lipids and blood sugar, etc. 23, 24, 25. Atractylenolides-I (AT-I), AT-II, and AT-III are lactone constituents extracted from AM 26. Studies have confirmed that AT-I significantly inhibited cell proliferation and glycolysis and promoted apoptosis in colorectal cancer cells by inhibiting AKT/mTOR and JAK2/STAT3 signaling pathways, respectively 27, 28. Moreover, the anti-cancer effect of AT-II against chemo-resistance of colorectal cancer cells was related to the down-regulation of lncRNA XIST and ROR1, and the up-regulation of miR‐30a‐3p 29. An additional study showed that AT-III contributed to apoptosis of colorectal cancer via increasing the expression of Bax, cleaved caspase-3, and p53 as well as down-regulating the expression of Bcl-2 *in vitro* and *in vivo* 30*.* However, the current researcher's findings demonstrate that a great deal of the investigations currently remain in the early stages of cellular experiments. Most importantly, the effects of atractylenolides coupled with IFN-γ in CRC have yet to be addressed, and further investigations on animal experiments and even clinical trials are required to elucidate the scientific connotation of its clinical application.

In this study, we aimed to investigate atractylenolides and IFN-γ in combination to inhibit colorectal cancer and evaluate whether they tend to coordinate to inhibit the invasion and metastasis of colorectal cancer cells. In particular, we also explored whether IFN-γ might enhance the anti-tumor effect of AT-II by inhibiting the activity of the NF-kB p65/PD-L1 signaling pathway, thereby providing a novel therapy strategy for the treatment of CRC.

## Materials and Methods

### Cell culture

Human metastatic colon cancer cell lines HT29 and HCT15 cells were purchased from iCell Bioscience Inc. (Shanghai, China). All cells were cultured in Dulbecco's Modified Eagle's Medium (DMEM, Gibco) supplemented with 10% fetal bovine serum (FBS, Hangzhou Sijiqing Biological Engineering Materials Co., Ltd. Zhejiang, China), 1% penicillin, and 1% streptomycin (Beyotime, Shanghai, China), and incubated in a humidified atmosphere with 37°C and 5% CO_2_.

### Cell counting Kit-8 (CCK-8) assay

HT29 and HCT15 cells were inoculated into 96-well plates at a density of 5×10^3^ cells/well and cultured for 24 h. After that, the medium was replaced with 100 μl fresh medium containing increasing concentrations of AT-I, AT-II, and AT-III [0, 2, 5, 10, 20, 40, 80, 100, 200, and 400 μmol/L, dissolved in 0.1% dimethyl sulfoxidedmwas (DMSO)], respectively. The cells were then cultured for another 24 hours before being tested for vitality using a CCK-8 kit (Beyotime) in accordance with the manufacturer's instructions. Additionally, HT29 and HCT15 cells were cultured in DMEM containing different concentrations of AT-II (40, 80, 100, 200, and 400 μmol/L) and IFN-γ (62.5, 125, 250, 500, and 1000 ng/ml, dissolved in 0.1% DMSO) used alone or in combination for 24 and 48h. Subsequently, 10 μL cck-8 solution was added to each well and incubated at 37 °C for 4 h. Next, the absorbance of each well at 450 nm was measured using a microplate reader (CMaxPlus, Molecular Devices). The potential synergistic, additive, or antagonistic effects of AT-II and IFN-γ were assessed derived from CCK-8. The combination index (CI) was calculated using CompuSyn software (Biosoft, UK) as described by Chou and Talalay 31. As a result, CI< 1, CI ±1, and CI>1 reflect synergy, additivity, and antagonism, respectively.

### Transwell migration and invasion assay

The effect of AT-II and IFN-γ on the migration capacity of HT29 and HCT15 cells was investigated utilizing transwell chambers. HT29 and HCT15 cells were added into the upper chamber of Transwell which is without Matrigel at a density of 5 × 10^4^ cells/mL in 100 μL serum-free DMEM, while 600 μl of DMEM containing 15% FBS were added to the lower chambers. AT-II (80 μmol/L as a final concentration) and IFN-γ (250 ng/ml) alone or in combination were added to the upper chamber, and the solutions were co-cultured for 24 h. After incubation for 24h, cells that migrated through the filter into the lower chamber were fixed by 4% paraformaldehyde (Shanghai Macklin Biochemical Co., Ltd, China) and stained by 0.1% crystal violet (Shanghai Qiangshun Chemical Reagent Co., Ltd, China) and counted under a light microscope (MOTIC CHINA GROUP CO., LTD. Nanjing, China). For the invasion assay, it was carried out in an operation as the migration assay, with the addition of a Matrigel (BD Biosciences, Billerica, MA, USA).

### *In vivo* experiments

To establish the CRC xenograft model and lung metastasis model, a total of 24 male BALB/c nude mice (5-6 weeks old, 16-19 g) and 24 male C57BL/6 mice were purchased from the Shanghai SLAC Laboratory Animal Co., Ltd (SCXK (Hu) 2017-0005, Shanghai, China)) and housed in specified pathogen-free (SPF) conditions with ad libitum food and water. For the xenograft CRC model, mice were injected subcutaneously with 100 μL of the HCT15 cell suspension at a concentration of 2×10^7^ cells/ml on the right flanks under an anesthetic with 3% isoflurane. During the tumor reached a size of approximately 100 mm^3^, mice were randomly divided into four groups (n = 6/group) that received daily intraperitoneal injections of normal saline, IFN-γ (0.3 mg/kg), AT-II (50 mg/kg) either alone or in combination for 21 days. During therapy, the tumor volumes were monitored every 3 days and calculated as (width^2^×length)/2. At the end of the experiment, the xenograft mice were sacrificed via inhalation of excess CO_2_. The tumor tissues were then collected and weighed. A section of tissues was fixed in 4% paraformaldehyde, paraffin-embedded, and sectioned for TdT-mediated dUTP nick end labeling (TUNEL) and one portion of the tumor tissues was used for flow cytometry analysis. For the lung metastasis model, the additional cohort of C57BL/6 mice were anesthetized and then 1×10^6^ HCT15 cells in 100 μL of phosphate-buffered saline were injected into the tail vein. After treatment of IFN-γ and AT-II alone or in combination for 28 days, the mice were euthanized, and the blood samples were collected and concentrated for cytokine analysis. Furthermore, lung tissues were obtained for metastatic nodule counting and hematoxylin-eosin (H&E) staining.

### TUNEL assay

Apoptotic cells in tumor tissues were investigated by using the One Step TUNEL Apoptosis Assay Kit (Red fluorescence) (C1090, Beyotime, China) according to the manufacturer's instructions. DAPI was employed to mark the nuclear area simultaneously. Images were acquired by a fluorescence inverted microscope (Olympus, Tokyo, Japan).

### Lung histopathological analysis

After 24 h fixation in 4% paraformaldehyde, the lung tissues acquired from C57BL/6 mice with pulmonary metastases were paraffin embedded for histopathological analysis. Next, the paraffin-embedded tissues were sectioned at 4 μm thick and then stained with hematoxylin and eosin. Finally, the images were taken using a light microscope (Nikon, Japan) at 200× magnification.

### Flow cytometry assay

Another part of the tumor tissues of lung metastatic C57BL/6 mice was collected for immune cell isolation. The tumor tissues were cut into tiny segments, followed by digestion in 10 mL digestion solution (Sigma-Aldrich) at 37 °C for 1h. The single-cell suspension was obtained by grinding digested tissues, followed by filtration using a 70-µm cell strainer (BD Biosciences, USA), and re-suspension of the cell precipitate in PBS after filtration. The Single-cell suspensions from tumor tissues were stained for 30 min using the following antibodies: Alexa Fluor® 488 Mouse Anti-Human CD3 (557694, BD Biosciences), FITC Mouse Anti-Human CD4 (556615, BD Biosciences), PE Mouse Anti-Human CD8 (555367, BD Biosciences), BV421 Mouse Anti-Human PD-L1 (568922, BD Biosciences), BV650 Mouse Anti-Human PD-1 (564324, BD Biosciences) on ice. Flow cytometry was carried out using a NovoCyte Advanteon flow cytometer (Agilent, USA) and results were analyzed using FlowJo software (Tree Star, Ashland, OR, USA).

### Enzyme-linked immunosorbent assay (ELISA)

The blood from lung metastatic C57BL/6 mice was collected and centrifuged at 3500 rpm at 4°C for 10 min to harvest serum. The supernatants collected were adopted to measure cytokines [IL-1α (P1561, Beyotime, China), IL-1β (ml063132, Shanghai Enzyme-linked Biotechnology Co., Ltd. China), IL-10 (MM-0176M1, Jiangsu Meimian Industrial Co., Ltd, China), IL-17 (MM-0170M2, Meimian, China), IL-2 (MM-0701M2, Meimian, China), IL-6 (MM-0163M1, Meimian, China), IL-12; P40 (ml037869, Shanghai Enzyme-linked Biotechnology Co., Ltd. China), Eotaxin (BMS6008, Thermo Fisher Scientific), CXCL1/KC/N51 (PC173, Beyotime, China), MCP-1 (MM-0082M2, Meimian, China), MIP-1β (ml037685, Shanghai Enzyme-linked Biotechnology Co., Ltd. China), and TNF-α (MM-0132M2, Meimian, China)] by commercial ELISA kits in accordance with the manufacturer's suggestions. The optical densities were measured on the CMaxPlus microplate reader (Molecular Devices) at 450 nm. The concentrations of cytokines were quantified in pg per ml.

### RNA isolation and real time-quantitative polymerase chain reaction (RT-qPCR)

Total RNA from the treated HT29 and HCT15 cells was extracted using TRIzol reagent (Thermo Fisher Scientific). Then, the concentration and purity of total RNA were analyzed by using spectrophotometry at 260/280 nm (Thermo Scientific, USA), and complementary DNA (cDNA) was synthesized from 1 μg of total RNA by PrimeScript™ RT reagent kit (Takara, Japan) at 37°C for 15 min and 85°C for 5 min. RT-qPCR was performed by using the SYBR Green qPCR Master Mix (Yeasen Biotechnology (Shanghai) Co., Ltd. China) with the LightCycler® 96 system (Roche, Switzerland) under conditions for 95°C for 10 min, 40 cycles of 95°C for 15 s, and 60°C for 30 s. The primers were synthesized by Sangon Biotech (Shanghai, China) and listed as follows: Slug: Forward 5′-CGAACTGGACACACATACAGTG-3′, Reverse 5′-CTGAGGATCTCTGGTTGTGGT-3′; Zeb1: Forward 5′-GATGATGAATGCGAGTCAGATGC-3′, Reverse 5′-ACAGCAGTGTCTTGTTGTTGT-3′; Foxc-1: Forward 5′-GGCGAGCAGAGCTACTACC-3′, Reverse 5′-TGCGAGTACACGCTCATGG-3′; Twist1: Forward 5′-GTCCGCAGTCTTACGAGGAG-3′, Reverse 5′-GCTTGAGGGTCTGAATCTTGCT-3′; β-actin: Forward 5′-CATGTACGTTGCTATCCAGGC-3′, Reverse 5′-CTCCTTAATGTCACGCACGAT-3′. β-actin served as an internal control. The expression levels of targets were quantified using the 2^-∆∆Ct^ method 32.

### Western blotting assay

Total proteins from the HT29 and HCT15 cells treated with AT-II, IFN-γ, p38 MAPK inhibitor (SB203581), and Wnt/β-catenin agonist (HLY78) alone or in combination were extracted by RIPA lysis buffer (Beyotime, China), and the protein concentration was measured using the bicinchoninic acid Protein assay kit (Beyotime) according to the manufacturer's instructions. Equal quantities (30 μg) of protein samples were separated by 8-12% sodium dodecyl sulfate-polyacrylamide gelelectrophoresis (SDS-PAGE) and then transferred onto polyvinylidene fluoride (PVDF) membranes (Millipore, USA). Membranes were blocked with 5% nonfat milk at room temperature for 1 h and washed three times, followed by incubation with primary antibodies against MMP2, MMP9, N-cadherin, E-cadherin, p-p38, P38, p-FAK, FAK, Wnt, β-catenin, Smad3, Smad1, p-NF-kB p65, NF-kB p65, and PD-L1 (Affinity Biosciences, Jiangsu, China) diluted at 1:1000 overnight at 4°C. β-actin (1:10000, Affinity) was used as the internal reference. The next day, the membranes were washed three times with TBST and incubated with the HRP-linked goat anti-rabbit IgG or HRP-linked goat anti-mouse IgG (1:6000, Cell Signaling Technology, Danvers, MA, USA) for 1 h at room temperature. The protein bands were visualized with the BeyoECL Plus kit and quantified by Image J software (v1.8.0, National Institutes of Health).

### Statistical analysis

Data are expressed as the mean ± standard deviations (SD). statistical analysis between groups was performed using one-way analysis of variance (ANOVA) and Student's *t-test*. Significance was *P<0.05*.

## Results

### Atractylenolides and IFN-γ suppressed the cell viability in CRC cells

**Figure 1:**
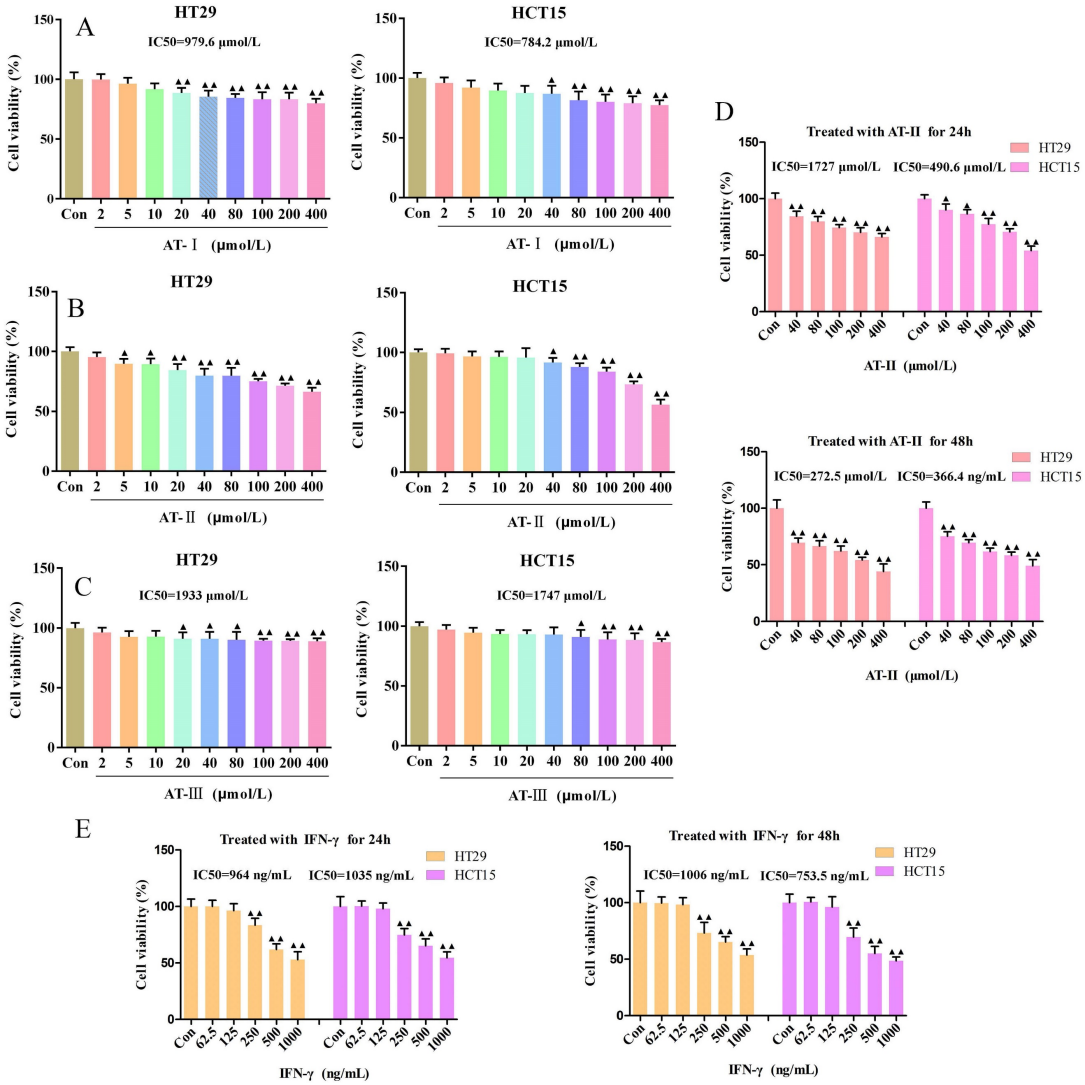
** Effect of atractylenolide-Ⅰ (AT-Ⅰ), AT-II, AT-III, and IFN-γ on cell viability in CRC cells.** HT29 and HCT15 cells were treated with AT-Ⅰ (A), AT-II (B), and AT-III (C) at 2, 5, 10, 20, 40, 80, 100, 200, and 400 μmol/L, respectively. Cell counting kit-8 (CCK-8) assay was used to measure the cell viability of HT29 and HCT15 cells. (D) CCK-8 assays were used to measure the cell viability of HT29 and HCT15 cells treated with AT-II at 40, 80, 100, 200, and 400 μmol/L for 24 and 48h, respectively. (E) Cell viability was measured by CCK-8 after HT29 and HCT15 cells were treated with IFN-γ at a dose of 62.5, 125, 250, 500, and 1000 ng/ml for 24 and 48h, respectively. Results were presented as mean ± SD, ^▲^*P<0.05*, ^▲▲^*P<0.01*, *vs*. Control (Con) group.

The proliferative effects of AT-I, AT-II, and AT-III at appointed concentrations (0, 2, 5,10, 20, 40, 80, 100, 200, 400 µM) on HT29 and HCT15 cells and AT-II (0, 40, 80, 100, 200, 400 µM) for different treatment times (24h and 48h) were observed by CCK-8 assays, respectively. The IC50 of AT-I, AT-II, AT-III, and IFN-γ is 979.6, 784.2; 1727, 490.6; 1933, 1747 µM; 964, 1035 ng/mL for HT29 and HCT15 cells, respectively (Figure 1A-1D). Results showed that AT-I, AT-II, and AT-III significantly inhibited cell viability in a dose-dependent manner in HT29 and HCT15 cells, especially with regard to AT-II. Similarly, a CCK-8 assay was employed to assess the effect of IFN-γ on the cell viability of HT29 and HCT15 cells. As shown in Figure 1D-1E, the cell viability was significantly inhibited by AT-II and IFN-γ treatment in time- and dose-dependent manners. These results suggested that atractylenolides and IFN-γ markedly suppressed hyper-proliferation of HT29 and HCT15 cells and 80, 100, and 200 µM of AT-II combined with 250, 500, 1000 ng/ml IFN-γ were chosen for the subsequent experiments.

### IFN-γ promotes the inhibitory effect of AT-II on cell proliferation in CRC cells

**Figure 2:**
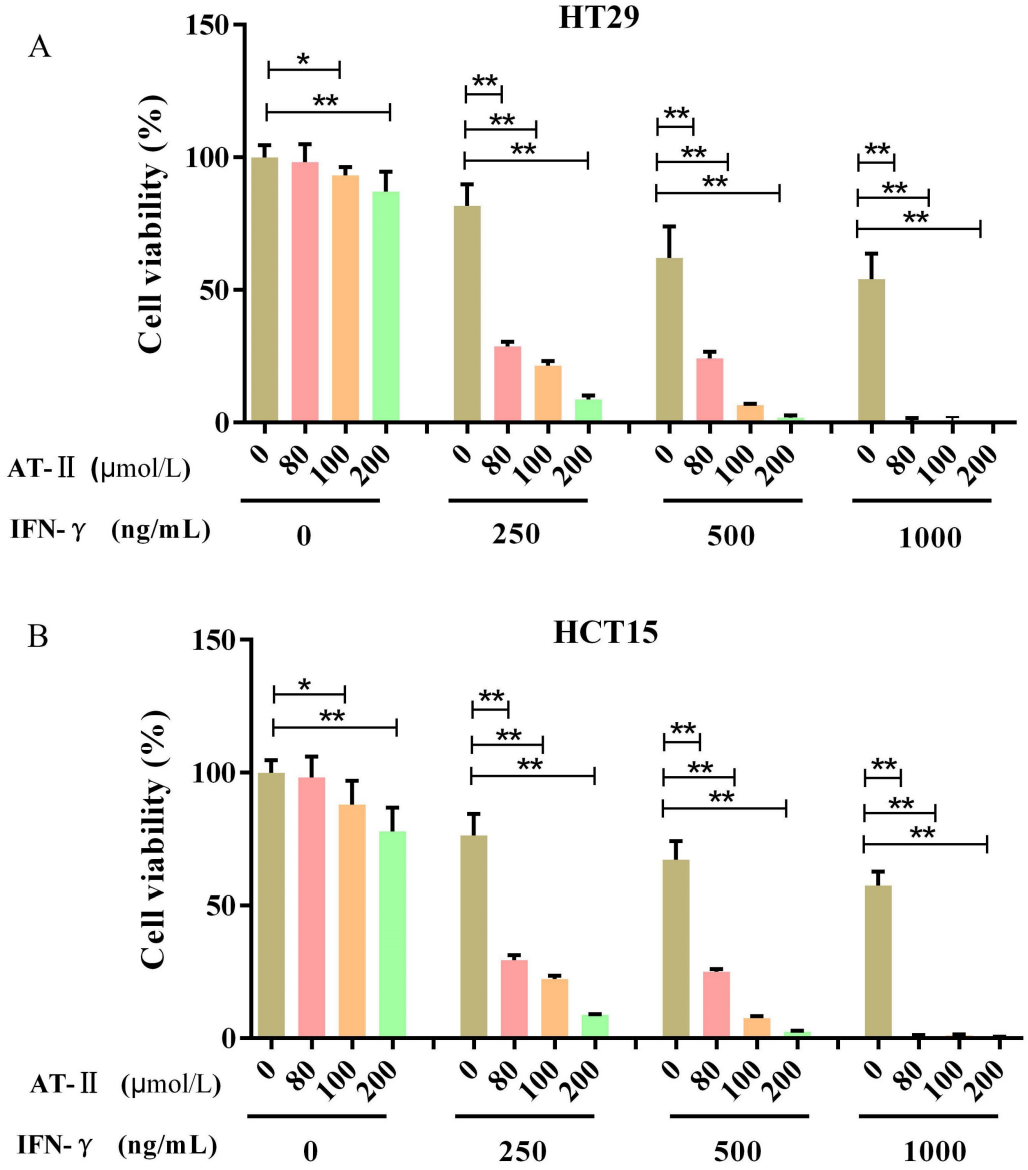
** IFN-γ strengthens the suppressive effect on cell growth of AT-II in CRC cells.** (A) HT29 and (B) HCT15 cells were administrated with increasing dosages of AT-II and IFN-γ alone or in combination. Then, the cell viability of HT29 and HCT15 cells was measured using CCK-8 assay after 24h. Data was expressed as mean ± SD. ^*^P<0.05, ^**^P<0.01, *vs*. Control (0) group.

HT29 and HCT15 cells were maintained with increasing concentrations of AT-II and IFN-γ alone or in combination. The cell proliferation was examined after 24h by CCK-8 assay to investigate whether IFN-γ might coordinated the effect of AT-II in HT29 and HCT15 cells (Figure 2A-2B). The acquired results were also used in CompuSyn software to evaluate the synergistic or additive effect exhibited by IFN-γ when coupled with AT-II intervention. The combination indices (CI) computed in HT29 and HCT15 cells were found to be much lower than the line of additivity (CI ≤ 1), revealing a synergistic influence even at the lowest mixture dosages (80 µM AT-II and 250 ng/ml IFN-γ) given in combination (Table 1). The result permitted us to employ smaller amounts of both compounds, which gave us the equivalent inhibition obtained by combining the two supplements in therapies with a single medication. Therefore, all of those that followed combined interventions of each medicine were used at the lower concentration providing CI<1, in particular, HT29 and HCT15 cells were cultured with 80 μmol/L of AT-II and 250 ng/ml of IFN-γ, treated independently or in combination.

### AT-II combined with IFN-γ inhibited migration and invasion of CRC cells

**Figure 3:**
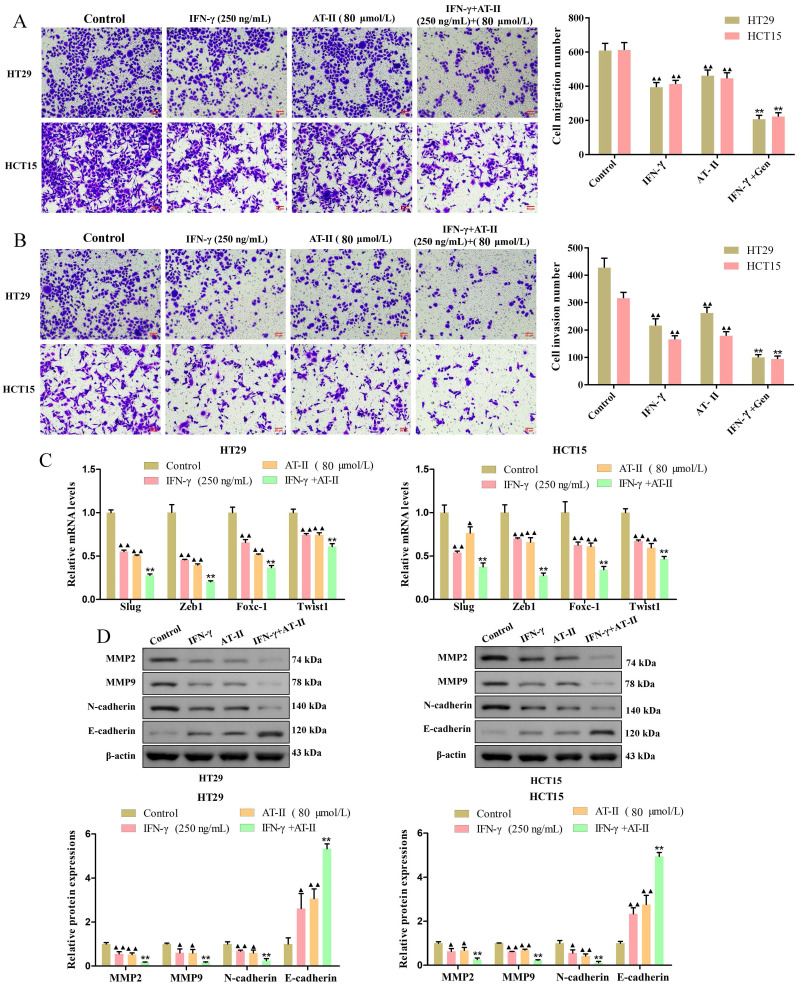
** IFN-γ cooperates with AT-II suppressed migration and invasion of CRC cells.** (A-B) Transwell assays showed that the combination of IFN-γ and AT-II significantly inhibited the migration and invasion of HT29 and HCT15 cells. Scale bar, 50 μm. (C) The levels of migration-associated genes slug, zeb1, foxc-1, and twist1 in CRC cells were assessed using qRT-PCR. (D) Protein expression levels of migration and invasion-related proteins MMP2, MMP9, N-cadherin, and E-cadherin were detected by Western blotting analysis. All the data are shown as the mean ± SD. ^▲^*P<0.05*, ^▲▲^*P<0.01*, *vs*. Control group. ^*^P<0.05, ^**^P<0.01, *vs*. IFN-γ group.

Transwell migration and invasion assay measured the transfer performance of the HT29 and HCT15 cells. The results revealed that the treatment of AT-II and IFN-γ alone significantly inhibited the migration and invasion of CRC cells. As expected, there were significant differences between AT-II and IFN-γ groups and IFN-γ in combination with AT-II, the cell migration and invasion number of AT-II combined with IFN-γ treatment groups were lower than that of alone groups (Figure 3A-3B). The qRT-PCR results showed that the expression of invasion-associated targets slug, zeb1, foxc-1, and twist1 was effectively down-regulated in HT29 and HCT15 cells treated with IFN-γ and AT-II alone or in combination (Figure 3C). Moreover, the results of the Western blotting showed that treatment of IFN-γ and AT-II alone or in combination decreased MMP2, MMP9, and N-cadherin protein levels while increased E-cadherin protein levels in HT29 and HCT15 cells (Figure 3D). Together, these findings revealed that a combination of IFN-γ and AT-II could significantly inhibit the migration and invasion of HT29 and HCT15 cells.

### AT-II combined with IFN-γ attenuated p38 MAPK, FAK, Wnt/β-catenin, and Smad pathways in CRC cells

**Figure 4:**
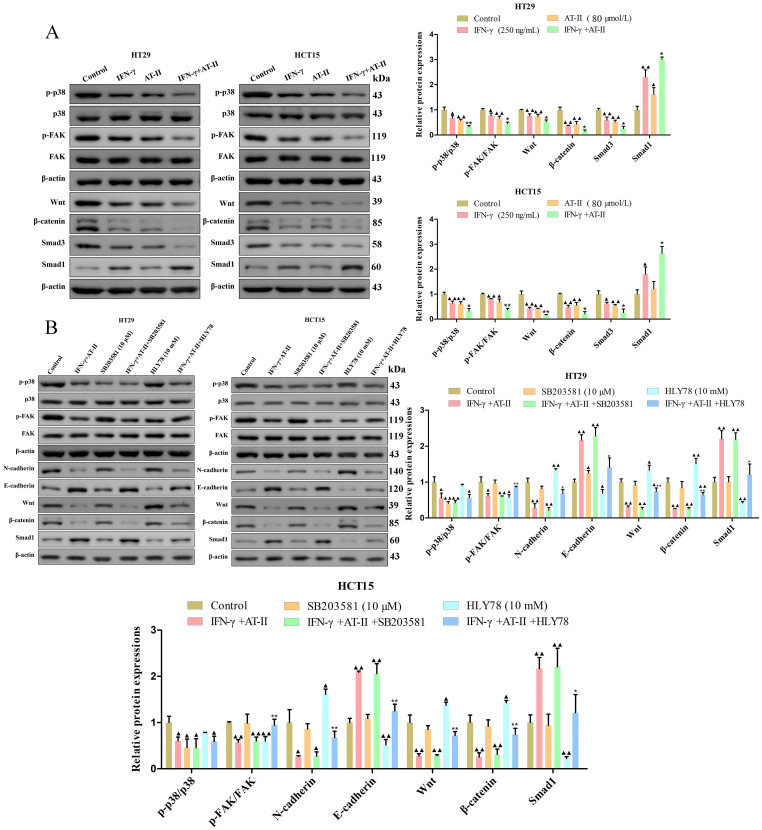
** The activity of FAK/p38 MAPK, Wnt/β-catenin, and Smad pathways in CRC cells interfered with either IFN-γ, AT-II, SB203581, or HLY78 alone or in combination.** (A) Western blot analysis of p-p38, p38, p-FAK, FAK, Wnt, β-catenin, Smad3, and Smad1 levels in HT29 and HCT15 cells treated with IFN-γ or AT-II alone or combination. (B) Western blot for the FAK/p38 MAPK, Wnt/β-catenin, and Smad-related protein expression. FAK/p38 MAPK, Wnt/β-catenin, and Smad-related proteins were reversely upregulated by HLY78 (Wnt/β-catenin pathway activator). Results were shown as mean ± SD. ^▲^*P<0.05*, ^▲▲^*P<0.01*, *vs*. Control group. ^*^P<0.05, ^**^P<0.01, *vs*. IFN-γ+AT-II group.

For the p38 mitogen-activated protein kinases (p38 MAPK, p38), Focal adhesion kinase (FAK), Wnt/β-catenin, and Smad signaling pathways are connected with the progression of cell proliferation, migration, and invasion in cancers 33-36. The results of the western blot demonstrated that AT-II combined with IFN-γ suppressed the phosphorylation of p38 and FAK. Furthermore, the expression of Wnt, β-catenin, and Smad3 decreased and that of Smad1 increased were observed in both AT-II combined with IFN-γ treated HT29 and HCT15 cells (Figure 4A). To further identify the essential role of p38 and Wnt/β-catenin in AT-II+IFN-γ-induced inhibition of migration in both HT29 and HCT15 cells, SB203581, as a p38 inhibitor and HLY78, a Wnt/β-catenin agonist was used to prove the aforementioned hypothesis. It showed that the expression levels of FAK-, EMT-, and Smad-related proteins were reversed by SB203581 and HLY78, as illustrated in Figure 4B. Collectively, these results indicated that AT-II+IFN-γ inhibited the cell migration of HT29 and HCT15 cells was induced by p38 and Wnt/β-catenin-dependent pathway for regulating the phosphorylation of FAK and upstream signaling pathway of Smad1.

### AT-II combined with IFN-γ inhibited NF-kB p65/PD-L1 pathway in CRC cells

**Figure 5:**
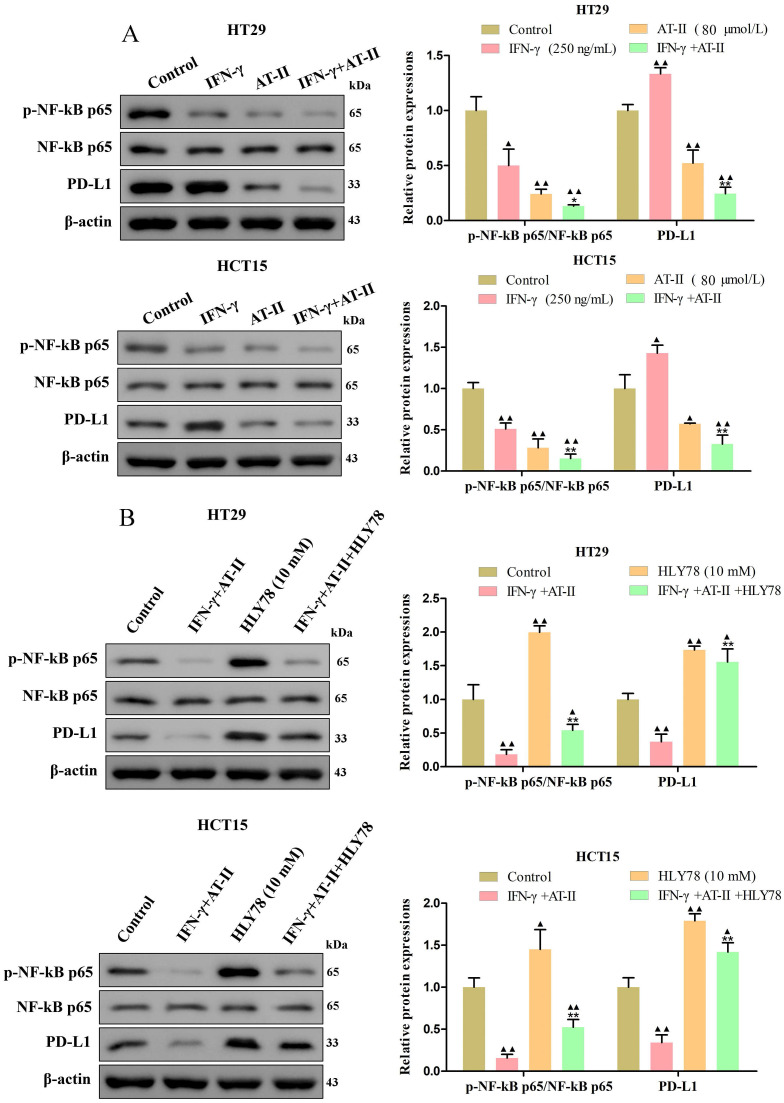
** The activity of NF-kB p65/PD-L1 pathway in CRC cells treated with either IFN-γ, AT-II, or HLY78 alone or in combination.** (A) Western blot analysis of p-NF-kB p65, NF-kB p65, and PD-L1 in HT29 and HCT15 cells treated with IFN-γ, AT-II or combination. (B) Western blot analysis of p-NF-kB p65, NF-kB p65, and PD-L1 levels in HT29 and HCT15 cells treated with combinations of IFN-γ, AT-II, and HLY78. Results were presented as mean ± SD. ^▲^*P<0.05*, ^▲▲^*P<0.01*, *vs*. Control group. ^*^P<0.05, ^**^P<0.01, *vs*. IFN-γ+AT-II group.

PD-L1 is dysregulated in several malignancies and is required for immune escape 37. Previous studies showed that NF-kB p65 binding to the PDL1 promoter contributes to stimulating the expressions of PD-L1 38, 39. As shown in Figure 5A, we found that the expression of the p-NF-kB p65 and PD-L1 was significantly reduced in HT29 and HCT15 cells treated with IFN-γ and AT-II alone or in combination compared to control. Similarly, we further confirmed that the effect of AT-II combined with IFN-γ on the expression of p-NF-kB p65 and PD-L1 was significantly attenuated under the treatment of HLY78 (Figure 5B). Together, these results illustrated that AT-II combined with IFN-γ regulated phosphorylation of NF-kB p65 to mediated expression levels of PD-L1 in CRC cells.

### AT-II combined with IFN-γ results in apoptosis and inhibition of metastasis *in vivo*

**Figure 6:**
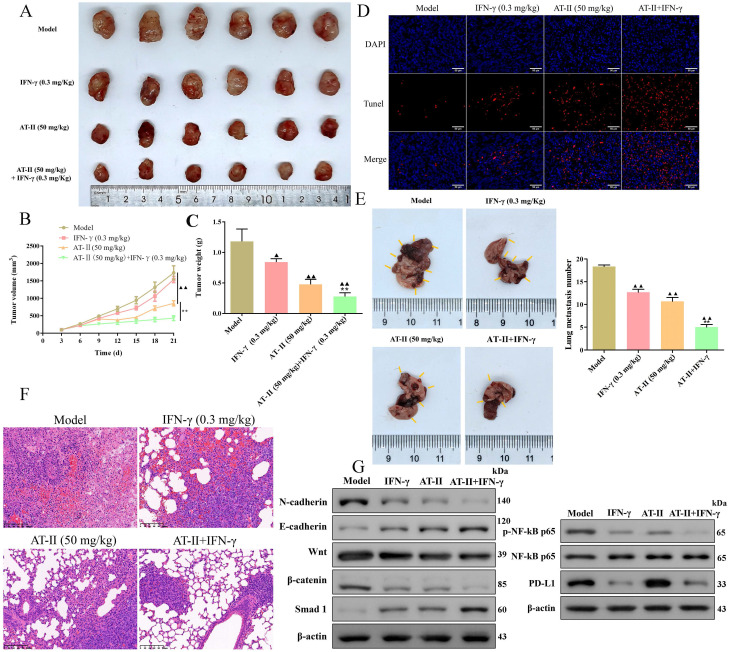
** AT-II combined with IFN-γ suppressed growth and lung metastasis* in vivo*.** (A) Images of the xenograft tumors from BALB/c (nu/nu) mice after treatment of IFN-γ or AT-II or combination. (B) Tumor volume and (C) tumor weights xenograft tumors in each group. (D) Tunel staining showing the levels of cell apoptosis in xenograft tumors. Scale bar, 50 µm. HCT-15 cells were injected into another cohort of C57BL/6 mice through the caudal vein, and lung metastasis was assessed after treatment of IFN-γ or AT-II or a combination for 30 days. (E) Image of lung tissues in C57BL/6 mice in each group, and the statistical analysis of lung metastasis number was performed at the experimental endpoint. (F) H&E staining of the lung tissues in lung metastatic specimens. Scale bar = 100 μm. (G) Western blot analysis showing the expression of N-cadherin, E-cadherin, Wnt, β-catenin, Smad1, p-NF-kB p65, NF-kB p65, and PD-L1 proteins in lung tissues of lung metastatic C57BL/6 mice after treatment of IFN-γ or AT-II or combination. ^▲^*P<0.05*, ^▲▲^*P<0.01*, *vs*. model group. ^**^P<0.01, *vs*. IFN-γ and AT-II groups.

To investigate the therapeutic potential of IFN-γ and AT-II used alone or in combination* in vivo*, an HCT15 tumor-bearing xenograft mice and a lung metastasis C57BL/6 mice model were established, respectively. Consistently, AT-II alone and AT-II combined with IFN-γ treatment resulted in a remarkably decreased tumor volume (Figure 6A-6B) and weight (Figure 6C). Moreover, Tunel assays showed that IFN-γ and AT-II used alone or in combination evidently increased the apoptosis of the tumor cells of subcutaneous tumor tissues in HCT15 tumor-bearing mice (Figure 6D). As delineated in Figure 6E, the metastatic lung nodules were decreased in IFN-γ, AT-II, and AT-II+IFN-γ groups compared to those in the model group. The haematoxylin and eosin analysis revealed that AT-II combined with IFN-γ treatment remarkably decreased the infiltration by tumor cells in the lung tissues of metastatic C57BL/6 mice (Figure 6F). We further examined the EMT biomarkers, Wnt/β-catenin, Smad, and NF-kB p65/PD-L1 pathways by Western blotting. As shown in Figure 6G, AT-II combined with IFN-γ obviously suppressed the N-cadherin, Wnt, β-catenin, p-NF-kB p65, and PD-L1 of lung tissues, it also increased E-cadherin and Smad1 expressions compared that with the model group. These results suggested that AT-II combined with IFN-γ is a potentially effective anticancer strategy for restraining proliferation and metastasis in CRC *in vivo* by targeting Wnt/β-catenin and NF-kB p65/PD-L1 pathways.

### AT-II combined with IFN-γ induces anti-tumor immunity *in vivo*

**Figure 7:**
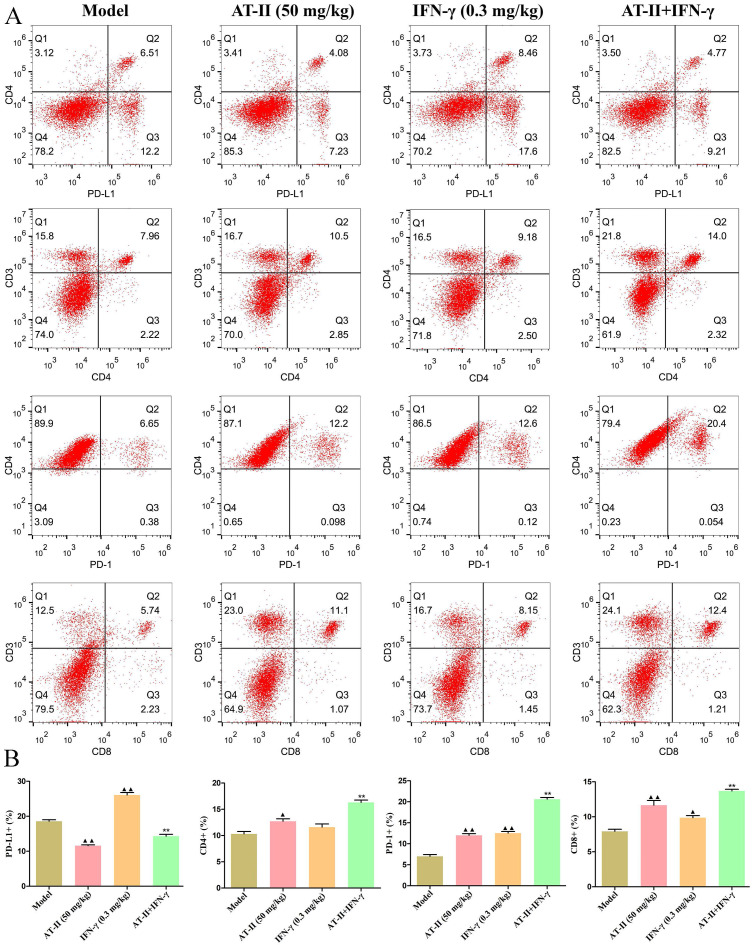
** Effect of AT-II combined with IFN-γ on the immune microenvironment *in vivo*.** (A) Representative images and (B) statistical results of PD-L1, CD4, PD-1, and CD8 expression in the tumour population of lung metastatic C57BL/6 mice are shown as indicated by flow cytometry. ^▲^*P<0.05*, ^▲▲^*P<0.01*, *vs*. model group. ^**^P<0.01, *vs*. IFN-γ and AT-II groups.

**Figure 8:**
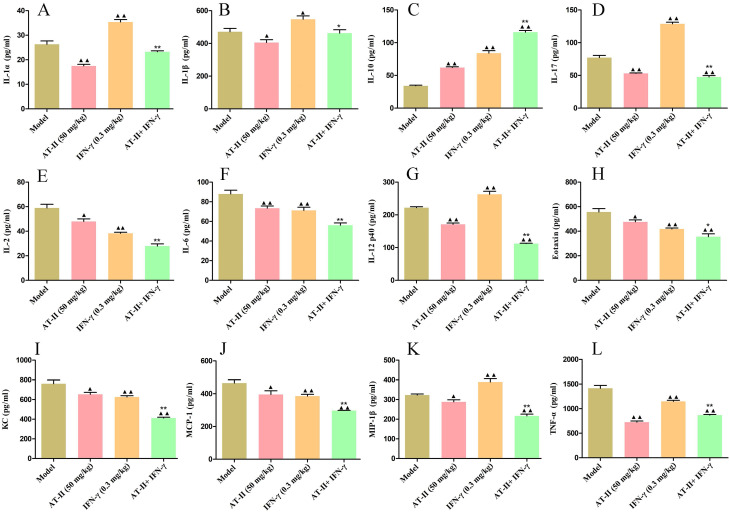
** Effect of AT-II combined with IFN-γ on cytokine profiling in peripheral blood of lung metastatic C57BL/6 mice.** The levels of (A) IL-1α, (B) IL-1β, (C) IL-2, (D) IL-6, (E) IL-10, (F) IL-12 (p40), (G) IL-17, (H) Eotaxin, (I) KC, (J) MCP-1, (K) MIP-1β, and (L) TNF-α in the serum samples from lung metastatic C57BL/6 mice were measured by using ELISA. Data were represented as mean±SD. ^▲^*P<0.05*, ^▲▲^*P<0.01*, *vs*. model group. ^**^P<0.01, *vs*. IFN-γ and AT-II groups.

To further evaluate the effects of intraperitoneal injection of AT-II combined with IFN-γ on anti-tumor immunity, on day 21 after the AT-II combined with IFN-γ treatment we detected PD-L1^+^, CD4^+^, PD-1^+^, and CD8^+^ cells by flow cytometry (Figure 7A). As shown in Figure 7B, a significant increase in the proportion of PD-L1^+^ cells in tumor tissues of lung metastatic C57BL/6 mice with IFN-γ treatment, whereas AT-II treatment significantly decreased the percentage of PD-L1^+^ cells singly or in combination. Furthermore, the proportion of CD4^+^, PD-1^+^, and CD8^+^ T cells dramatically increased in the tumor microenvironment after the AT-II combined with IFN-γ treatment. As illustrated in Figure 8A-8L, compared to the model group, the content of tumor immunity-related cytokines IL-1α, IL-1β, IL-2, IL-6, IL-12 (p40), IL-17, Eotaxin, KC, MCP-1, MIP-1β, and TNF-α decreased, while IL-10, associated with promoting anti-tumor immunity, increased in the serum of lung metastatic C57BL/6 mice. Overall, these results suggest that AT-II combined with IFN-γ activates the anti-tumor immune responses in the tumor microenvironment *in vivo*.

## Discussion

CRC is the most frequent malignant tumor of the digestive system, with a significant morbidity and fatality rate. Furthermore, up to 50% of patients present with tumor metastases at the time of their initial diagnosis 40. Currently, the available therapeutic options include surgery, systemic chemotherapy combined with biologically targeted therapy, and immunotherapy, but the high expense of treatment, its side effects, and complications caused an increasing economic burden on health in advanced CRC patients.

With the lightning-fast growth of TCM in recent years, TCM combined with surgery, chemotherapy, radiotherapy, immunotherapy, and cytokine therapy have come to be available, all of which are capable of minimizing metastasis and recurrence of CRC as well as postoperative adverse reactions, lowering patient mortality and improving quality of life 41. As a result, the application of monomers and compound Chinese medicinal preparation according to the theory of TCM might provide novel strategies for the clinical treatment of CRC. Previous studies confirmed that the rhizome of *Atractylodes macrocephala* Koidz could be used to treat different cancers by regulating tumor cell immunity 42, 43. In this study, we found that AT-I, AT-II, and AT-III inhibited the viability of HT29 and HCT15 cells, particularly for AT-II. To the best of our knowledge, IFN-γ, a cytokine synthesized and secreted by activated immune cells in the tumor microenvironment, is inextricably linked with the inhibition of tumor cells. In addition, we found that AT-II and IFN-γ significantly inhibited cell viability in HT29 and HCT15 cells in a time and dose-dependent manner. Moreover, dose sensitivity experiments were performed for both AT-II and IFN-γ treated alone or in combination. The CCK-8 assays evidently suggested that a low concentration of IFN-γ (250 ng/mL) strengthened AT-II-triggered proliferation restraint. Here, the CI values estimated in HT29 and HCT15 cells were discovered to be substantially below the line of additivity (CI ≤ 1), demonstrating a synergistic effect for even the smallest amount of the two drugs (80 µM AT-II and 250 ng/mL IFN-γ) supplied in combination.

For the beginning of metastasis, cancer cells undertake a phenotypic transition from epithelial to mesenchymal cells, which is commonly referred to as EMT 44. In general, the decrease of E-cadherin expression and the elevation of N-cadherin were found in a series of metastatic tumors that contributed to the migration and invasion of cancer cells. Besides, the MMP-2 and MMP-9 are primarily accountable for the extracellular matrix (ECM) degradation and impairment of the basement membrane 45. In this study, our results suggested that AT-II and IFN-γ used alone or in combination diminished cell migration, invasion, and EMT through up-regulation of E-cadherin and down-regulation of N-cadherin, as well as promoted ECM degradation via inhibition of MMP2 and MMP9 in HT29 and HCT15 cells. Therefore, these results indicate that AT-II combined with IFN-γ may serve as a therapeutic strategy in treating metastatic CRC.

There is strong evidence to suggest that the p38 MAPK pathway could be involved in cell proliferation, angiogenesis, cell invasion, and metastasis in tumorigenesis 46, 47. Zhang *et al.* suggested that apigenin combined with chrysin obviously decreased cell migration and invasion ability while increasing the cell apoptosis in SW480 and HCT-116 cells by trimming the activation of the p38-MAPK/AKT pathway 48. Cell migration is a multistage, highly interconnected process that begins with the protrusion of the cell surface and focal adhesions. FAK, an important factor, is responsible for cancer invasion and metastasis by inducing the expression of MMP2 and MMP9 in CRC 49. Furthermore, Wnt/β-catenin and Smad pathways play a vital role in the later stages of invasion and metastasis of CRC 50, 51. Importantly, a study by Lucas *et al.* suggested that the induction of PD‑L1 was transcriptionally mediated by NF-κB in colorectal cancer cells 52. In our study, the western blotting assay showed that AT-II combined with IFN-γ significantly inhibited the p38, FAK, Smad, Wnt/β-catenin, and NF-κB p65/PD-L1 signaling pathways. To detect whether p38 MAPK and Wnt/β-catenin pathway was associated with the suppressive effect of the combination of AT-II and IFN-γ in CRC cells, the alterations of corresponding targets were observed after p38 inhibitor or Wnt/β-catenin agonist addition in HT29 and HCT15 cells. Our data showed that SB203581, a p38 inhibitor, didn't enhance AT-II and IFN-γ treatment caused decreases of p-P38, p-FAK, N-cadherin, and Wnt/β-catenin levels. Meanwhile, the results supported that HLY78, Wnt/β-catenin agonist, reversed the effects of AT-II and IFN-γ treatment on p38 MAPK, FAK, Wnt/β-catenin, Smad, and NF-κB p65/PD-L1 pathways. These results revealed that AT-II combined with IFN-γ treatment showed an interaction effect on cell proliferation and migration of HT29 and HCT15 cells through partly regulating the Wnt/β-catenin pathway.

**Figure 9:**
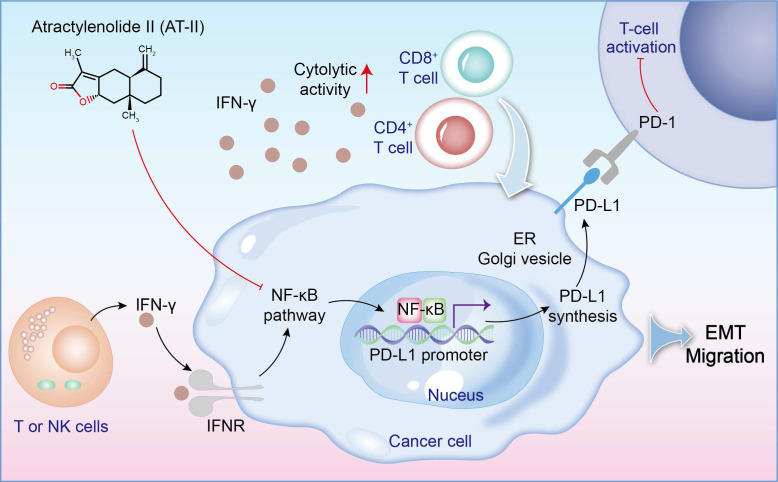
A schematic of the effect of AT-II combined with IFN-γ on NF-kB p65/PD-L1 pathway in colorectal cancer metastasis.

In order to assess the role of AT-II combined with IFN-γ on tumor growth *in vivo*, in our study, the subcutaneous xenotransplanted tumor mice and lung metastasis model of CRC were used. We found that AT-II combined with IFN-γ significantly inhibited tumor growth and lung metastasis in CRC mice, as accompanied by inactivation of Wnt/β-catenin and NF-κB p65/PD-L1 signaling. Additionally, we also observed that AT-II combined with IFN-γ significantly increased CD4^+^ T and CD8^+^ T cell infiltration in lung metastatic C57BL/6 mice. With the advancement of tumor experiments, an extensive amount of *in vivo* and *in vitro* studies have demonstrated that inflammatory cytokines may promote tumor cell metastasis by influencing the tumor microenvironment, boosting invasion capabilities, and minimizing immune cell function 53, 54. In this study, we found that AT-II combined with IFN-γ decreased the content of IL-1α, IL-1β, IL-2, IL-6, IL-12 (p40), IL-17, Eotaxin, KC, MCP-1, MIP-1β, and TNF-α, while increased the levels of IL-10 in the serum of HCT15 tumor-bearing C57BL/6 mice with lung metastases. Generally, our results showed that combination therapy with AT-II and IFN-γ down-regulated PD-L1 and inflammatory cytokines and increased PD-1 expression and the infiltration of CD4^+^/8^+^ T cells, indicating that combination therapy could effectively restore the T cell functions by attenuating anti-tumor immunological effect of CRC (Figure 9).

## Conclusions

In summary, these results showed that combination therapy with AT-II and IFN-γ dramatically inhibited tumor growth and lung metastasis, while ameliorating the immune microenvironment of CRC by regulating the expression of Wnt/β-catenin and NF-κB p65/PD-L1 signaling. Taken together, we suggested that combining AT-II and IFN-γ is an exciting possibility in the realm of immunotherapeutic medicines, but further extensive laboratory studies are needed to completely confirm this intriguing combination.

## Figures and Tables

**Table 1:** Combination index (CI) values calculated for each combined treatments in HT29 and HCT15 cells

HT29			HCT15		
AT-II (μmol/L)	IFN-γ (ng/mL)	CI	AT-II (μmol/L)	IFN-γ (ng/mL)	CI
80	250	0.52	80	250	0.41
100	250	0.62	100	250	0.5
200	250	0.61	200	250	0.46
80	500	0.77	80	500	0.58
100	500	0.54	100	500	0.37
200	500	0.43	200	500	0.3
80	1000	0.25	80	1000	0.18
100	1000	0.39	100	1000	0.39
200	1000	0.02	200	1000	0.14

## Abbreviations

CRC: Colorectal cancer
IFN-γ: Interferon (IFN)-γ
AT-II: Atractylenolides-II
CCK-8: Cell Counting Kit-8
ELISA: Enzyme-linked immunosorbent assay
IL: Interleukin
GM-CSF: Granulocyte-macrophage-colony stimulating factor
PD-L1: Programmed death-ligand 1
PD-1: Programmed death 1
TCM: Traditional Chinese medicine
AM: Atractylodes macrocephala
CI: Combination index
TUNEL: TdT-mediated dUTP nick end labeling
H&E: Hematoxylin-eosin
cDNA: complementary DNA
SDS-PAGE: Sodium dodecyl sulfate-polyacrylamide gelelectrophoresis
PVDF: Polyvinylidene fluoride
SD: Standard deviations
ANOVA: one-way analysis of variance
FAK: Focal adhesion kinase
ECM: Extracellular matrix
